# Supplementary material for: Brain responses vary in duration-modeling strategies and challenges
Source: Imaging Neurosci (Camb). 2025 Nov 10;3:IMAG.a.1003. doi: 10.1162/IMAG.a.1003 (PMC12603654; doi:10.1162/IMAG.a.1003)
Supplement: Supplementary Materials [file IMAG.a.1003_supp.pdf]

Supplemental File A

A.1 Noise Preprocessing

Eyes closed resting state data was preprocessed as follows

1. Data was down-sampled to 500Hz
2. EEGLAB's `pop_clean_rawdata()` was used to reject bad channels using Parameters: `FlatlineCriterion = 5`; `ChannelCriterion = 0.8`; `LineNoiseCriterion = 4`; `Highpass = [0.25, 0.75]`
3. Data was re-referenced to average reference
4. A copy of the data was made, and the copy was high-pass filtered at 1.5Hz
5. ICA was calculated on the (filtered) copied data
6. Bad components were marked using ICLabel with Parameters only set for eye movements and muscle artefacts (i.e. only eye- and muscle-artefacts were rejected)
7. ICA weights and reject-markers were copied to original data and bad components were subtracted
8. Rejected channels were interpolated via spherical interpolation

Additionally, during simulations the data was down-sampled (default 100Hz) and high-pass filtered (default 0.5 Hz) to match the simulations.

A.2 No Noise Results

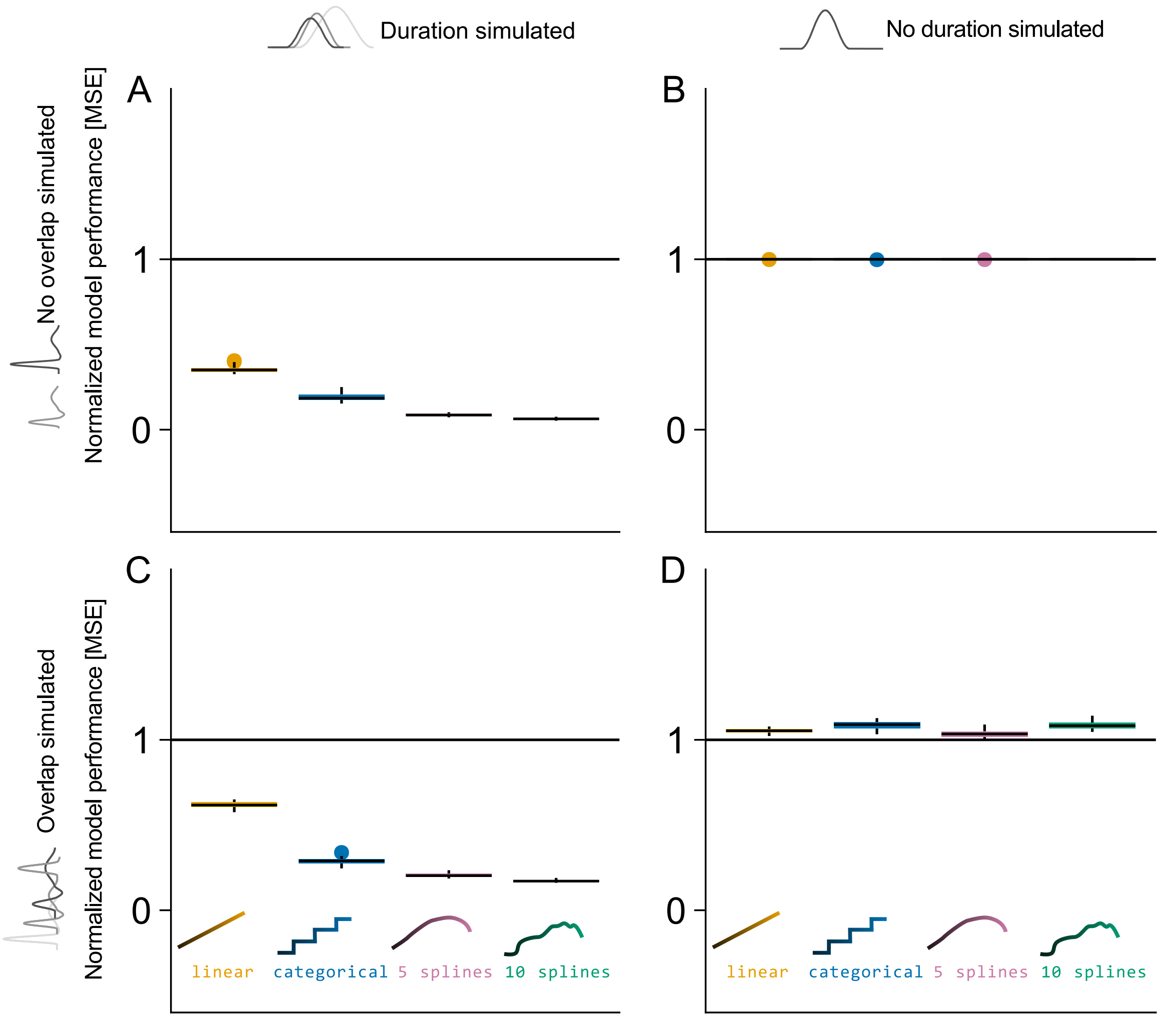

Figure S.1: Normalized mean squared error results for the four tested models (including duration as: linear, categorical, 5-spline, 10-spline variable) in different simulation settings. Black line (y-value one) indicated results from classical averaging; MSE of zero indicated perfect estimation of the ground truth. (A) Results when a duration effect, but no overlap is simulated. The spline strategies outperform the other strategies. (B) Results when no duration effect and no overlap were simulated, but duration effects were still estimated. Little overfit is visible here. (C) Results when duration effects were simulated, signals overlap, and overlap correction was used for modeling. No interaction between duration modeling and overlap correction was observed on the MSE performance. (D) Results when duration effect was not simulated, and signals overlap, and results are overlap-corrected.

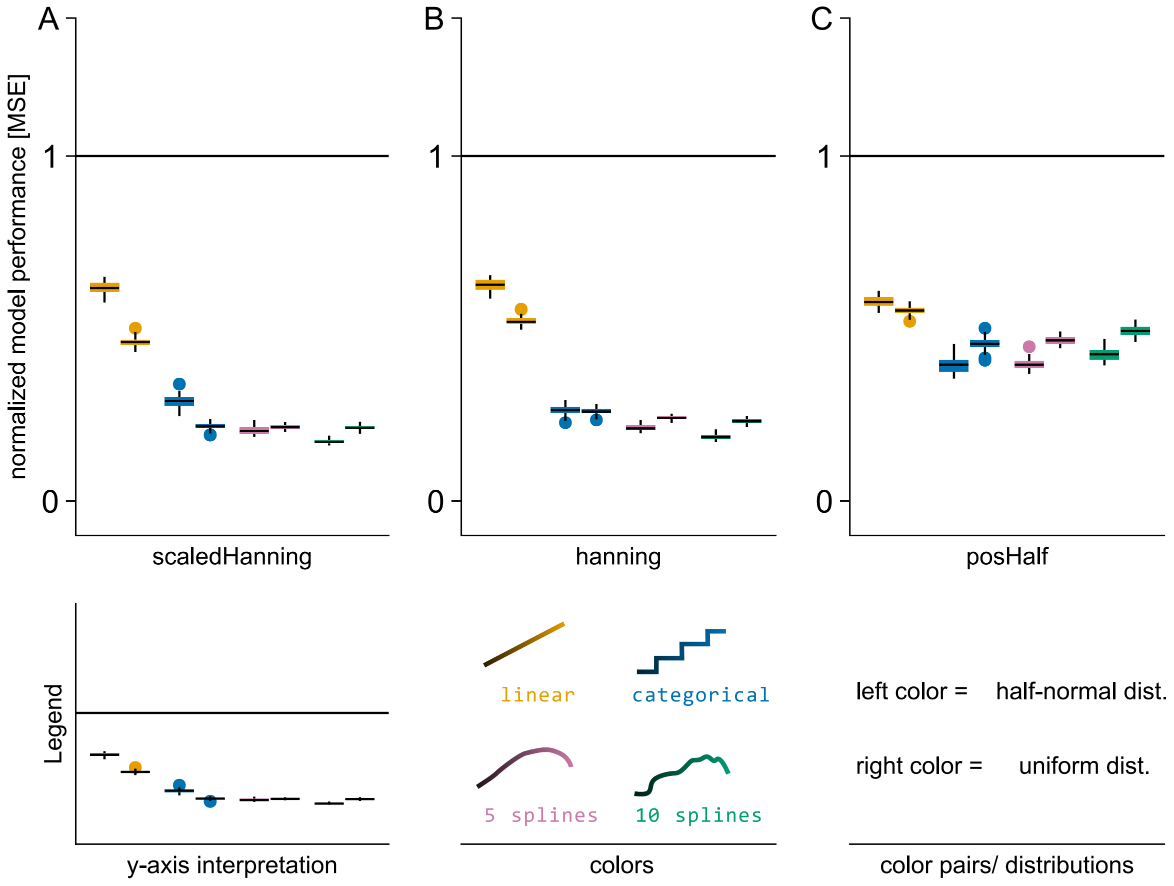

1112 Figure S.2: Normalized mean squared error results for the four tested models in simulations
1113 without noise (including duration as: linear, categorical, 5-spline, 10-spline variable) between
1114 shapes (A-C) and duration distributions (color pairs). Black line (y-value one) indicated
1115 results from classical averaging; MSE of zero indicated perfect estimation of the ground truth.
1116 Parameter settings for all panels: duration affects shape; overlap simulated; overlap-corrected/
1117 modeled.

A.3 Duration Modeling vs. Overlap Correction

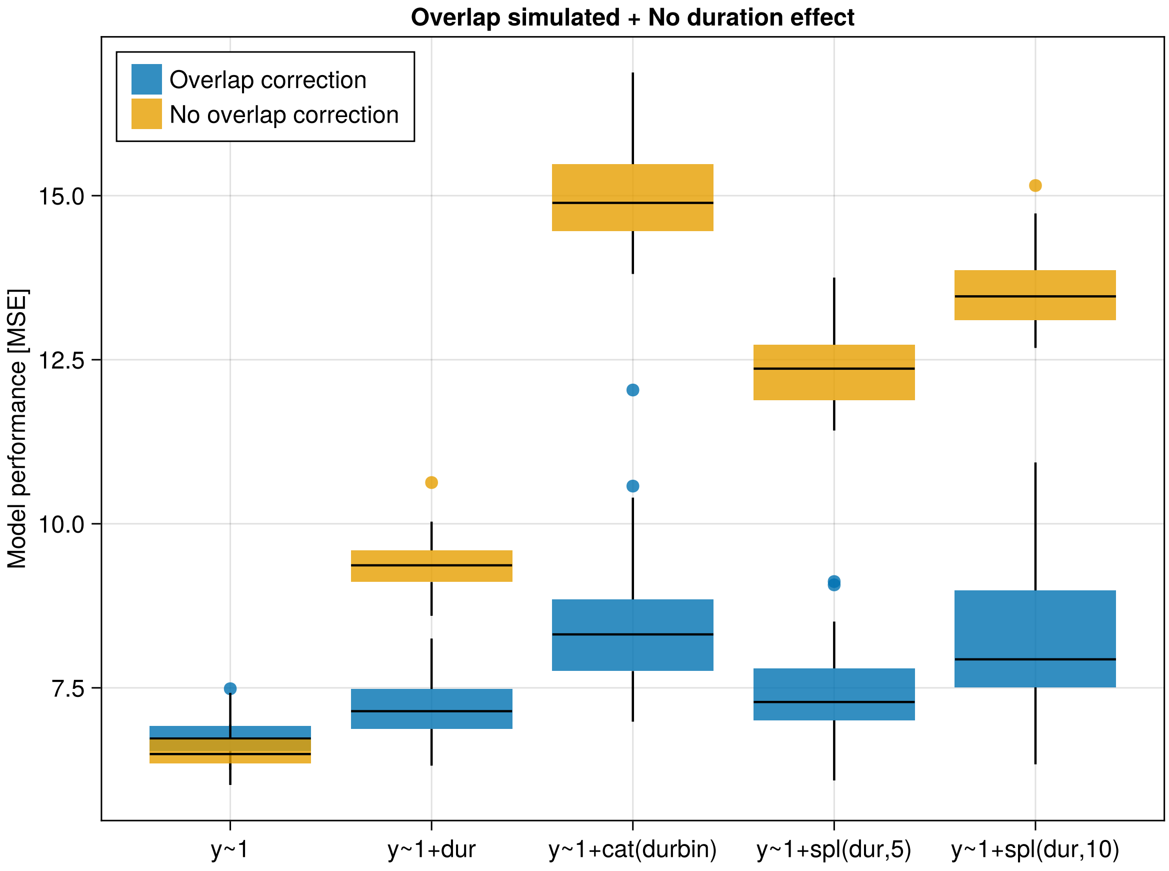

Figure S.3: *Not* normalized mean square error results when overlap is simulated, but no duration effect is simulated for five different models (classical averaging ($y \sim 1$) plus the four tested models (duration as: linear, categorical, 5-spline, 10-spline variable)), once with overlap correction (blue) and once without overlap correction (orange).

A.4 MSE Plots of Baseline Corrected Data

Baseline was corrected for each marginalized ERP independently in with a baseline period of −0.5s to 0.0s.

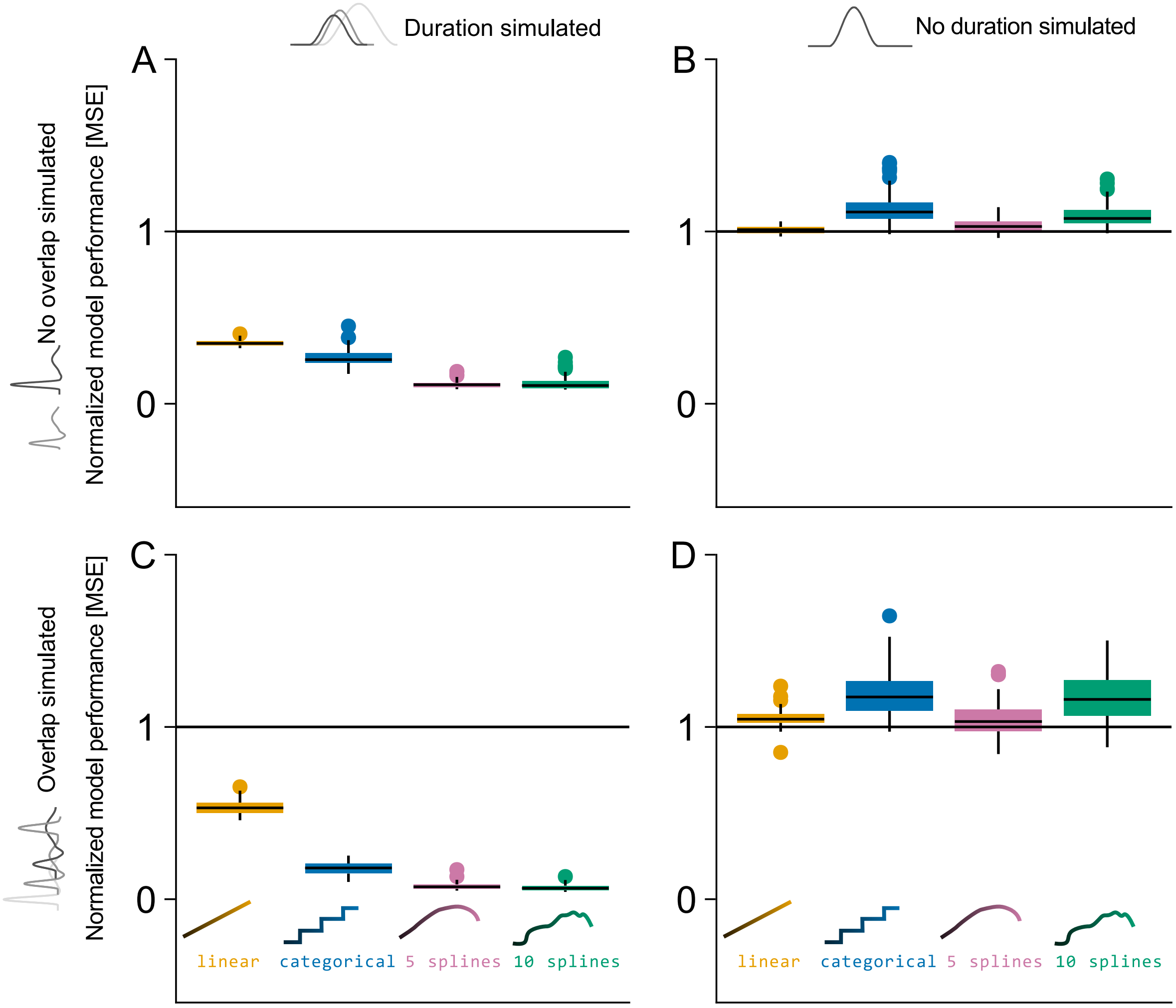

Figure S.4: Normalized mean squared error results <u>of baseline corrected estimates</u> for the four tested models (including duration as: linear, categorical, 5-spline, 10-spline variable) in different simulation settings. Black line (y-value one) indicated results from classical averaging; MSE of zero indicated perfect estimation of the ground truth. (A) Results when a duration effect, but no overlap is simulated. The spline strategies outperform the other strategies. (B) Results when no duration effect and no overlap were simulated, but duration effects were still estimated. Little overfit is visible here. (C) Results when duration effects were simulated, signals overlap, and overlap correction was used for modeling. No interaction between duration modeling and overlap correction was observed on the MSE performance. (D) Results when duration effect was not simulated, and signals overlap, and results are overlap-corrected.

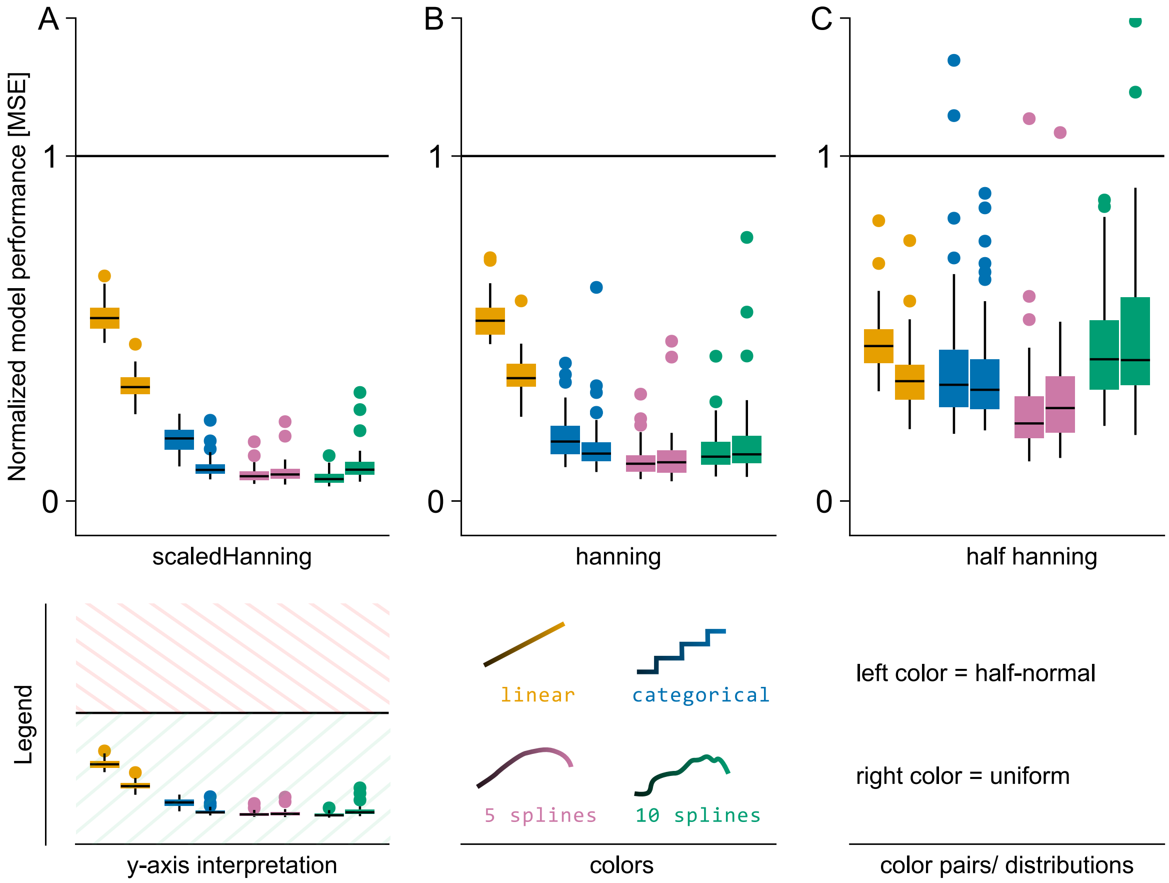

Figure S.5: Normalized mean squared error results of baseline corrected estimates for the four tested models in simulations without noise (including duration as: linear, categorical, 5-spline, 10-spline variable) between shapes (A-C) and duration distributions (color pairs). Black line (y-value one) indicated results from classical averaging; MSE of zero indicated perfect estimation of the ground truth. Parameter settings for all panels: duration affects shape; overlap simulated; overlap-corrected/ modeled.

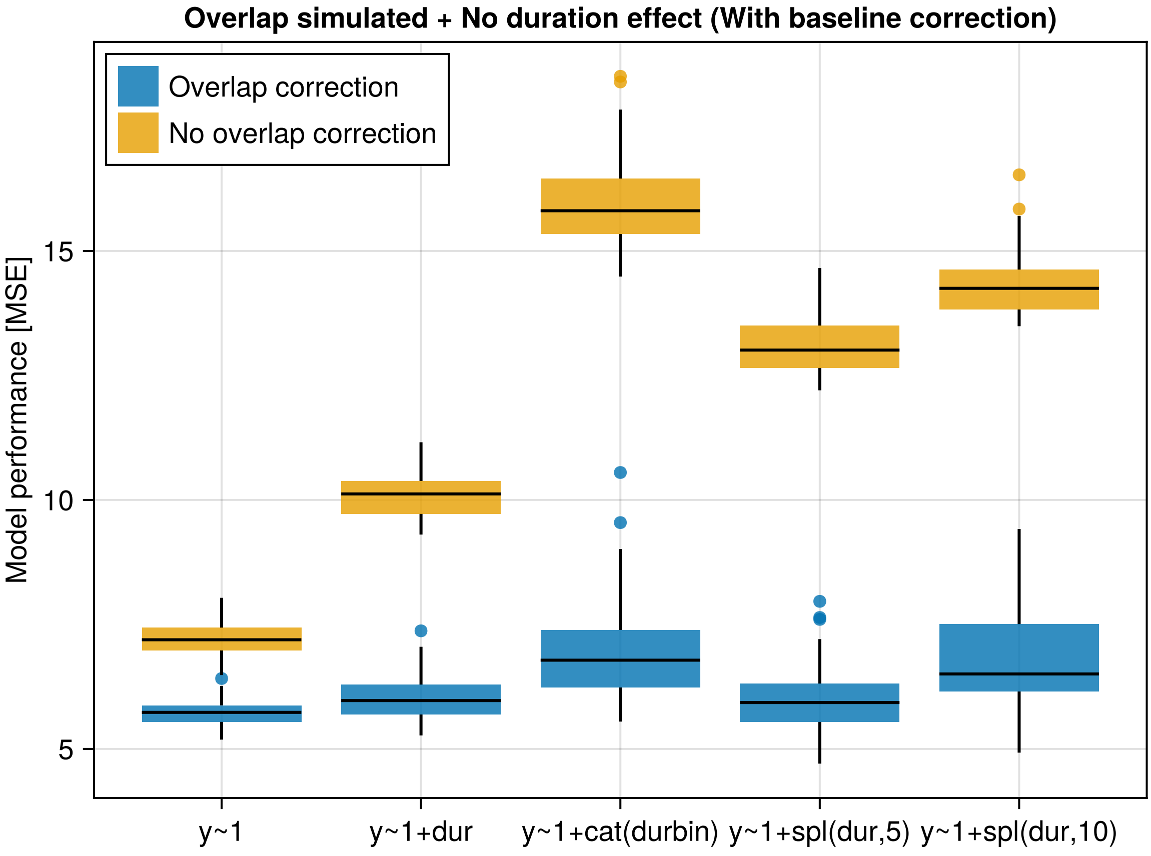

Figure S.6: *Not* normalized mean square error results of baseline corrected estimates when overlap is simulated, but no duration effect is simulated for five different models (classical averaging ($y \sim 1$) plus the four tested models (duration as: linear, categorical, 5-spline, 10-spline variable)), once with overlap correction (blue) and once without overlap correction (orange).

A.5 Alternative visualization of results from one simulation

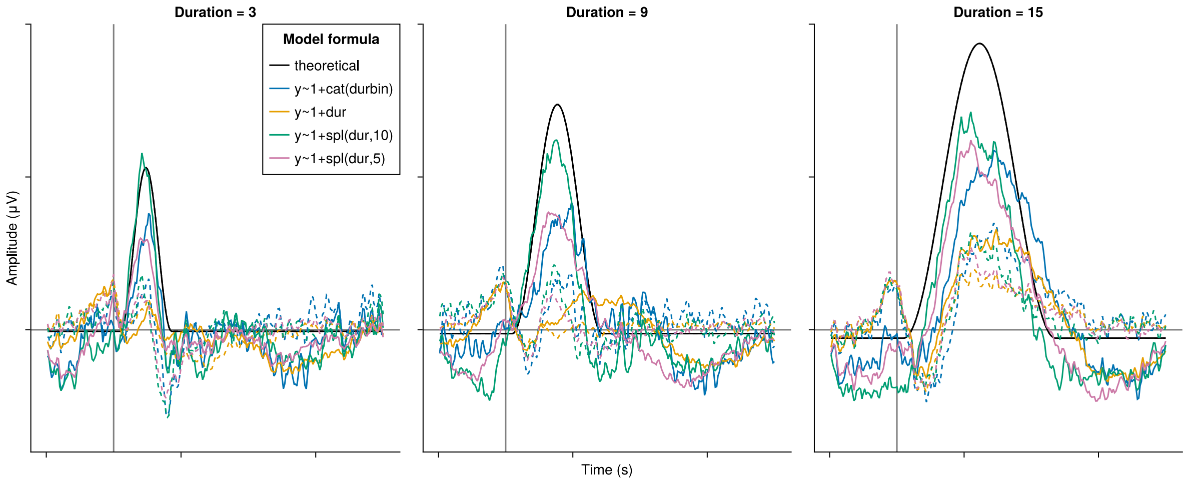

Figure S.7: Alternative version of Figure 4 for model comparison. Here, each panel corresponds to one of three specific durations (out of 15 marginalized effects), and all models (distinguished by color) are superimposed onto the ground truth (black line). Solid lines indicate overlap corrected models; dashed lines indicate not overlap corrected models.

A.6 Alternative reaction time standardization during fMRI analysis

A.6.1 80% Winsorization

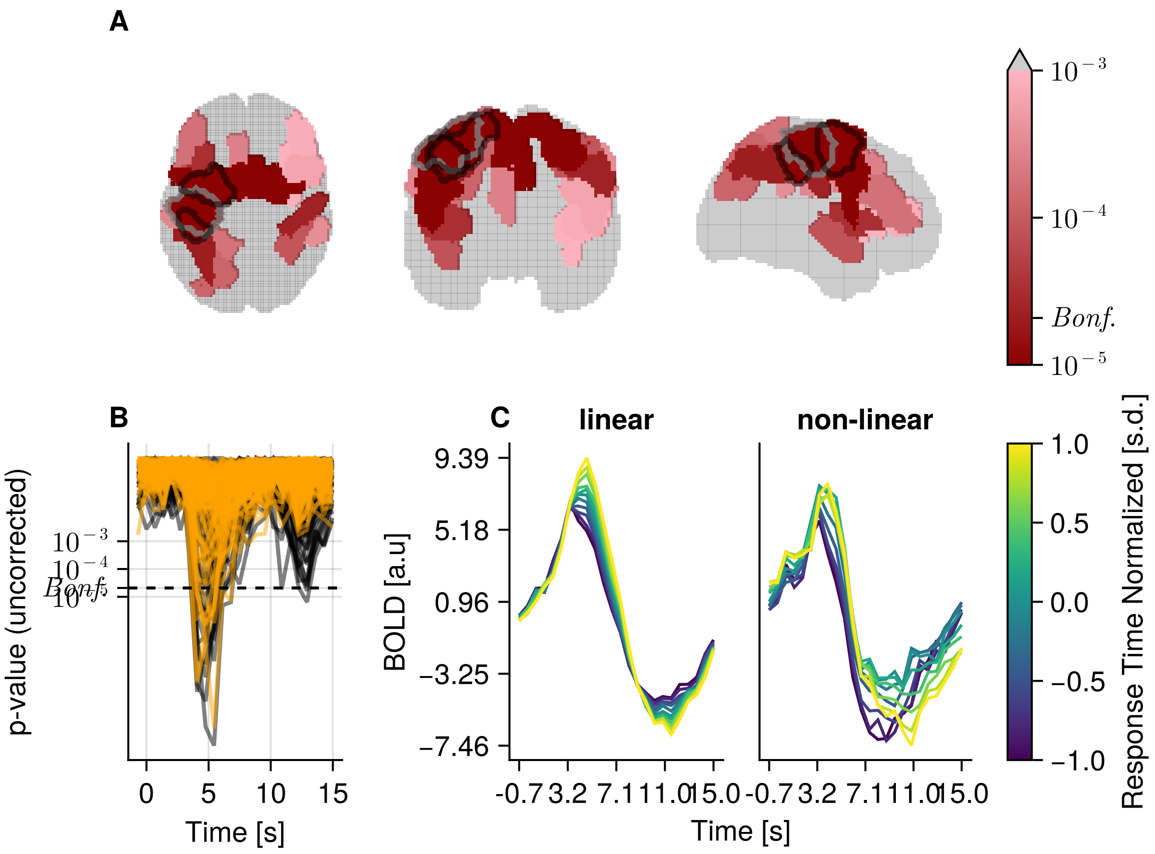

Figure S.8: fMRI results *after a reaction time standardization with a more stringent setting of an 80% winsorization (i.e. the highest and lowest 10th percentile is winsorized; see Figure 9 & Section 4).* (A) One slice of the transverse, coronal, and sagittal planes with ROIs colored according to their minimal p-value over time, uncorrected. Grey areas indicate p-value $> 10^{-3}$. Indicated Bonferroni threshold was calculated over time and ROI ($\alpha = \frac{0.05}{24} x100$). Marked regions (black outline) were significant under FDR correction over time and ROI. (B) Uncorrected p-values over time for all ROIs, with a linear model (black) and non-linear model (orange). The dashed line indicates the cut off for p-values after Bonferroni correction. (C) Marginal effects of the estimated BOLD response of one example region (gray outlined in A), for the linear (left) and non-linear (right) model.

A.6.2 Median + Median Absolute Deviation

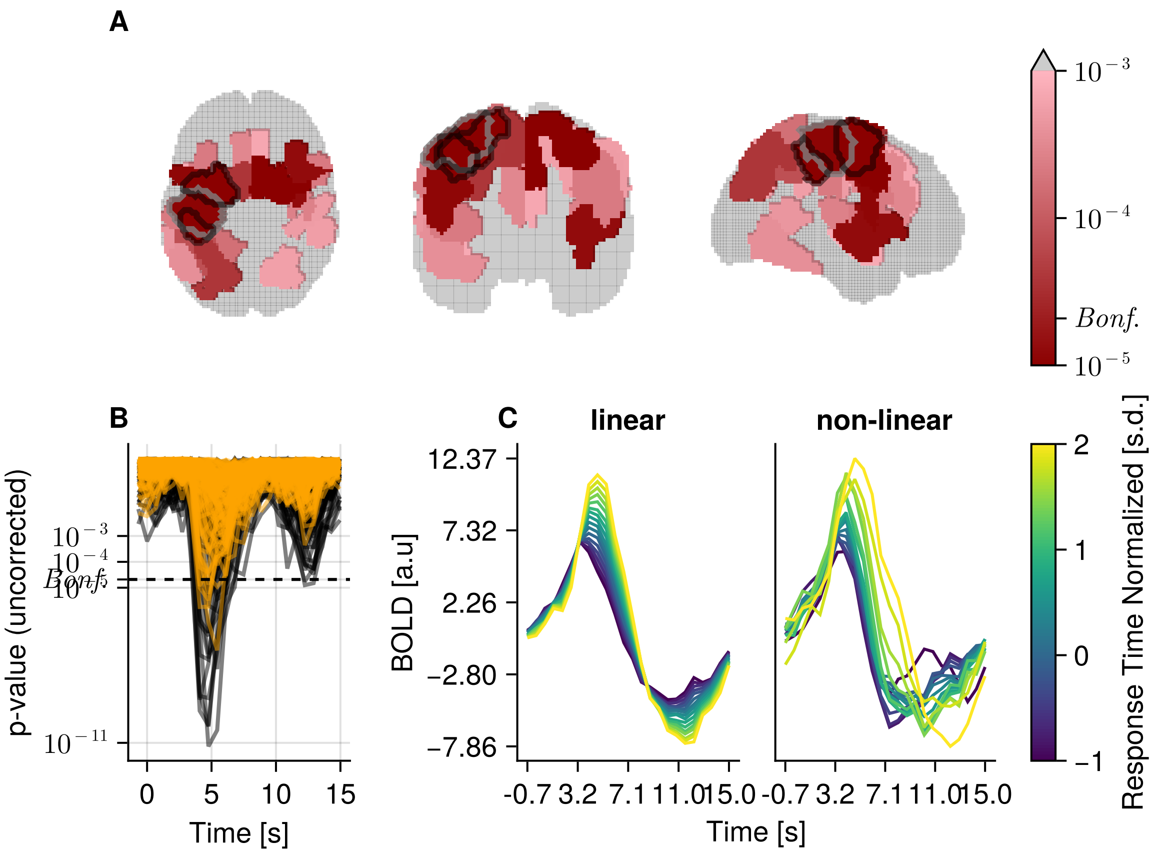

Figure S.9: fMRI results *after a reaction time standardization with a more robust measure and measure of spread: median and median absolute deviation; see Figure 9 and Section 4).* (A) One slice of the transverse, coronal, and sagittal planes with ROIs colored according to their minimal p-value over time, uncorrected. Grey areas indicate p-value $> 10^{-3}$. Indicated Bonferroni threshold was calculated over time and ROI ($\alpha = \frac{0.05}{24} x100$). Marked regions (black outline) were significant under FDR correction over time and ROI. (B) Uncorrected p-values over time for all ROIs, with a linear model (black) and non-linear model (orange). The dashed line indicates the cut off for p-values after Bonferroni correction. (C) Marginal effects of the estimated BOLD response of one example region (gray outlined in A), for the linear (left) and non-linear (right) model.

A.7 HRF Parameters

- p(1) - delay of response (relative to onset): 6
- p(2) - delay of undershoot (relative to onset): 16
- p(3) - dispersion of response: 1
- p(4) - dispersion of undershoot: 1
- p(5) - ratio of response to undershoot: 6
- p(6) - onset {seconds}: 0
- p(7) - length of kernel {seconds}: 32
